# Safety, Technical and Clinical Success of the Aperio Hybrid Thrombectomy Device in Acute Ischemic Stroke, a Prospective Post-market Clinical Follow-up Study (HYBRID)

**DOI:** 10.1007/s00062-025-01578-5

**Published:** 2025-10-23

**Authors:** Claudia Klüner, Benedikt Sundermann, Catalin George Iacoban, Daniel Behme, Kai Kallenberg, Olaf Wunderlich, Bernd Turowski, Wolfgang Reith, Thomas Liman, Thilo Rusche, Hannes Nordmeyer, Christian Mathys

**Affiliations:** 1 Institute of Radiology and Neuroradiology, Evangelisches Krankenhaus Oldenburg, Universitätsmedizin Oldenburg, Oldenburg, Steinweg 13–17, 26122 Oldenburg, Germany; 2 Research Center Neurosensory Science, Carl von Ossietzky Universität Oldenburg, Oldenburg, Germany; 3 Clinic of Radiology, Medical Faculty, University of Münster, Münster, Germany; 4 Clinic for Neuroradiology, Otto-Von-Guericke-University Magdeburg, Magdeburg, Germany; 5 Klinik für Diagnostische und Interventionelle Neuroradiologie, Klinikum Fulda, Fulda, Germany; 6 Institut für Diagnostische und Interventionelle Radiologie und Neuroradiologie, Klinikum Dresden, Dresden, Germany; 7 Department of Diagnostic and Interventional Radiology, Medical Faculty, Heinrich-Heine-University, Düsseldorf, Germany; 8 Clinic for Diagnostic and Interventional Neuroradiology, Saarland University Hospital, Homburg, Germany; 9 Department of Neurology, School of Medicine and Health Sciences, Carl von Ossietzky University of Oldenburg, Oldenburg, Germany; 10 Center for Stroke Research Berlin (CSB), Charité—Universitätsmedizin Berlin, Berlin, Germany; 11 German Center for Neurodegenerative Disease DZNE, partner site Berlin, Berlin, Germany; 12 Department of Neuroradiology, Municipal Hospital Solingen, Solingen, Germany; 13 School of Medicine, Department of Health, University of Witten/Herdecke, Witten, Germany

**Keywords:** Thrombectomy, Stentretriever, Acute ischemic stroke, Mechanical recanalization, Post-market clinical follow-up

## Abstract

**Purpose:**

Stentretrievers are key devices for endovascular treatment of acute ischemic stroke. Aim of this post market clinical follow-up study was to asses safety and outcomes of interventions using the APERIO® Hybrid/Hybrid^17|21^ thrombectomy device (AHD) in routine clinical use.

**Methods:**

We conducted a prospectively monitored, multicenter, national registry study with single-arm data collection including patients with acute intracranial vessel occlusion (anterior and posterior circulation, including medium vessel occlusions) who were treated with the AHD in Germany between November 2020 and June 2023. Patients (*n* = 173) with low pre-stroke morbidity (modified Rankin Scale [mRS] ≤ 2) were included. We assessed technical recanalization success (mTICI ≥ 2b), the occurrence of periprocedural symptomatic intracranial hemorrhages (sICH), good clinical outcome (mRS ≤ 2 at 90 days), and secondary outcomes.

**Results:**

Recanalization mTICI ≥ 2b was achieved in 84.4% with the AHD only, including 47.4% first pass success. Good clinical outcome at 90 days (mRS ≤ 2) was observed in 68.8%. Good primary safety outcome (no periprocedural sICH) was observed, notably even despite a high rate (79.8%) of stentretriever oversizing.

**Conclusion:**

The AHD is effective and safe. Technical success, primary safety and clinical outcomes surpassed earlier registries on different stentretrievers of the same and earlier generations. However, further studies are warranted to clarify whether these outcomes reflect true advantages of the device, improvements in thrombectomy setups and techniques, or selection biases.

**Supplementary Information:**

The online version of this article (10.1007/s00062-025-01578-5) contains supplementary material, which is available to authorized users.

## Introduction

After numerous studies have demonstrated high efficacy and good safety results for stentretriever-based endovascular stroke treatment, it is recognized as the standard procedure for recanalizing large vessel occlusions in patients with acute ischemic stroke (AIS) [1–7]. However, the standardization of both the procedure and the devices used remains limited.

The main tools used in endovascular stroke treatment, the stentretrievers, differ considerably in design [8]. While clinical outcomes do not vary substantially between conventional designs [9], innovative design features can have an impact on angiographic outcomes (such as distal embolization rates) [10]. Randomized studies with the purpose to compare different devices are rare [11], Post market clinical follow-up (PMCF) registries provide additional data on the safety and efficacy of devices in regular clinical use. PMCFs contribute to fulfilling regulatory requirements for continuous clinical surveillance [12].

Examples of larger registry studies on other stentretrievers are the TREVO Stent-Retriever Acute Stroke (TRACK) registry [13], the North American Solitaire Acute Stroke (NASA) registry [14] and the Embotrap Extraction & Clot Evaluation & Lesion Evaluation for NeuroThrombectomy (EXCELLENT) registry [15].

One aim of the design changes to stentretrievers was to improve their angiographic visibility [16] The APERIO® Hybrid/Hybrid^17|21^ thrombectomy device (AHD) is the successor of the Aperio stentretriever (Acandis, Pforzheim, Germany). The newer AHD (CE mark approval in 2019, no FDA approval) incorporates most features of the previous device version such as repeating functional segments of small closed cells and large open cells, aiming to improve integration and retention to the thrombus [17]. Additionally, it is equipped with embedded radiopaque drawn filled tubing (DFT), which allow full-length visibility of the device under fluoroscopy. Because the additional DFT wires increased the device profile, the initial version of the AHD had to be delivered through a 0.021 “microcatheter [17]. In a second step, a version with a lower profile, compatible with 0.017” microcatheters, was introduced for vessel diameters between 1.0–4.0 mm [18]. Early retrospective multicenter experience indicated a favorable safety profile and optimistic clinical results with the AHD [17–20], similar to the precursor device [19, 20]. A prospective registry (no monitoring) of anterior circulation strokes treated with the AHD with otherwise broad inclusion criteria confirmed these results [21]. Additionally, initial positive prospective results of using the AHD in distal intracranial occlusions have been reported (prospective REVISAR registry, congress presentation) [22]. Here we present the results of a prospective, monitored and multicentric PMCF study, collecting comprehensive information on technical and clinical success and safety of the use of the AHD in clinical practice. This study includes both anterior and posterior circulation strokes in patients with relatively low pre-stroke morbidity.

## Methods

### General Study Design

The HYBRID study (clinicaltrials.gov ID: NCT04457479) is a prospective, single arm, multicentric, national PMCF study. The study was approved by the medical ethics committee at the University of Oldenburg (coordinating review board) and subsequently by the local ethics committees at each site. The study was carried out in accordance with the Declaration of Helsinki. Patients or their legal guardians provided informed consent. In cases where consent was not available, e.g. because of death, the need for informed consent to analyze limited initial clinical data (anonymized) was waived. This was done in order to avoid selection biases, mainly due to early mortality after the endovascular procedure.

Originally, eight sites in Germany using or planning to use the AHD were asked to enter each patient treated with AHD into the registry. Seven sites actually included patients between 11/2020 and 06/2023. In the first part of the study, data was collected from routine clinical records in the treatment of patients with AIS using the AHD. The second part of the study consisted of a follow-up by face-to-face or telephone interview planned 90 days after the procedure to obtain information on the clinical outcome and any cerebrovascular events that had occurred in the meantime.

Assessment of clinical and image related data was carried out by study-related (neuro-)radiologists locally at each site. All study related data were reviewed by external monitors commissioned by the sponsor.

**Fig. 1:**
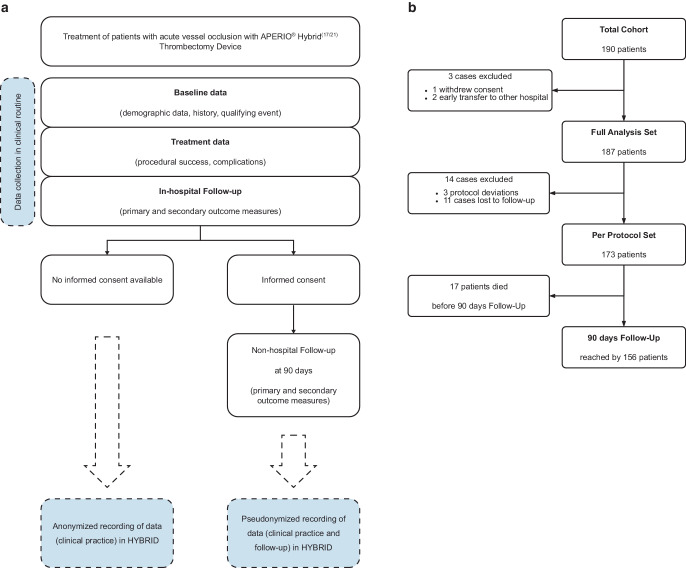
**a** Data acquisition flowchart. **b** Patient inclusion flowchart. Information on baseline clinical and imaging characterization, type of occlusion and treatment related are presented in Table 1 and in the more detailed supplementary Table S1

Pseudonymized data were collected for all patients after informed consent was obtained. In patients with no informed consent available, the follow-up interview after 90 days had to be omitted and anonymized data recording was performed. See Fig. 1a for an overview of data acquisition.

### In- and Exclusion Criteria

All patients treated with the AHD (all device sizes) according to the instructions for use (including deviations from the recommended stentretriever sizing) as a result of an AIS (inclusion criterion) were included in the study unless they met one of the exclusion criteria:

- patient age < 18 years [23], and/or
- pre stroke modified Rankin Scale [24, 25] (mRS) ≥ 3.

Screening logs were kept and monitored at each site for all patients meeting inclusion criteria, to ensure the most consecutive and unbiased patient inclusion possible.

### Primary Outcome Measures

#### The Three Primary Outcomes Comprised

- *Technical success*: Modified Thrombolysis in Cerebral Infarction [26] (mTICI) score ≥ 2b after treatment with AHD,
- *Good clinical outcome at 90 days:* mRS ≤ 2, and
- *Periprocedural symptomatic intracranial hemorrhage (sICH)*. The latter was defined as ICH in the postinterventional (< 24 h) CT associated with worsening of the National Institutes of Health Stroke Scale [27] (NIHSS, RRID:SCR_001804) by ≥ 4 points within 24 h [28, 29].

### Secondary Outcome Measures

Baseline characteristics typical for stroke-related studies were systematically recorded and can be found in Table 1 and in the more detailed supplementary Table S1.

**Table 1:** Summary of demographic and clinical characteristics as well as outcomes.

	HYBRID (*n* = 173)		TRACK (*n* = 629)		NASA (*n* = 354)	
	Data available (*n*)	*n* (%)	*n* (%)	*p* Value Hybrid vs. TRACK	*n* (%)	*p* Value Hybrid vs. NASA
*Demographics*						
Age [years], mean (SD)	172	73.2 (12.1)	66.1 (14.8)	< 0.0001	67.3 (15.2)	< 0.0001
Sex (female)	173	101 (58.4)	305 (48.3)	0.0212	176 (49.7)	0.0614
*Vascular risk factors*						
Arterial hypertension	173	131 (75.7)	473 (75.0)	0.8875	271 (76.6)	0.8332
Prior stroke	173	27 (15.6)	–	–	–	–
Artrial fibrillation	173	68 (39.3)	247 (39.2)	0.9928	148 (41.8)	0.5835
Diabetes mellitus	173	30 (17.3)	161 (25.5)	0.0240	87 (24.6)	0.0606
Hyperlipidemia	173	58 (33.5)	314 (49.8)	0.0001	182 (51.4)	0.0001
Coronary heart disease	173	28 (16.2)	146 (29.4)	0.0471	111 (31.4)	0.0002
Smoking history	173	38 (22.0)	154 (24.5)	0.4919	108 (30.5)	0.0396
*Medication*						
IV tPA	173	64 (37.0)	321 (51.2)	0.0011	–	–
*Clinical presentation*						
Initial NIHSS, median (IQR)	169	10 (5–17)	–	–	–	–
Initial NIHSS, mean (SD)	169	11.3 (7.4)	17.4 (6.7)	< 0.0001	18.1 (6.6)	< 0.0001
Initial systolic BP [mm Hg], mean (SD)	149	163.6 (29.0)	144.9 (26.6)	< 0.0001	–	–
Initial diastolic BP [mm Hg], mean (SD)	148	88.4 (18.4)	78.2 (19.2)	< 0.0001	–	–
*Admission type*						
In-house	173	2 (1.2)	–	–	–	–
Primary referral	173	130 (75.1)	–	–	–	–
Secondary referral	173	41 (23.7)	–	–	–	–
*Occlusion site*						
Left	173	73 (42.2)	–	–	–	–
Right	173	92 (53.2)	–	–	–	–
ACA	173	1 (0.6)	–	–	–	–
MCA	173	151 (87.3)	434 (68.9)	< 0.0001	197 (55.6)	< 0.0001
ICA	173	23 (13.3)	100 (15.9)	0.4000	82 (23.2)	0.0077
BA	173	7 (4.0)	–	–	36 (10.2)	0.0159
PCA	173	3 (1.7)	–	–	–	–
Anterior circulation	173	161 (93.1)	546 (86.7)	0.0241	–	–
Posterior circulation	173	12 (6.9)	84 (12.7)	0.0213	–	–
Vessel diameter (proximal thrombus boundary) [mm], mean (SD)	173	2.5 (0.8)	–	–	–	–
Vessel diameter (distal thrombus boundary) [mm], mean (SD)	173	1.8 (0.5)	–	–	–	–
Length of thrombus [mm], mean (SD)	173	9.7 (11.2)	–	–	–	–
More than one occlusion treated	173	15 (8.7)	–	–	–	–
*Infarction in stroke imaging (anterior circulation only; missing data in 3 patients)*						
ASPECTS 4	158	1 (0.6)	–	–	–	–
ASPECTS 5	158	4 (2.5)	–	–	–	–
ASPECTS 6	158	7 (4.4)	–	–	–	–
ASPECTS 7	158	21 (13.3)	–	–	–	–
ASPECTS 8	158	28 (17.7)	–	–	–	–
ASPECTS 9	158	37 (23.4)	–	–	–	–
ASPECTS 10	158	60 (38.0)	–	–	–	–
*Treatment*						
Number of passes (only 1st occl. evaluated, other devices included)						
1	172	95 (55.2)	272 (54.6)	0.0052	172 (48.6)	0.1527
2	172	39 (22.7)	167 (28.2)	0.3028	94 (26.6)	0.3369
3	172	20 (11.6)	112 (18.8)	0.0529	64 (18.1)	0.0581
> 3	172	18 (10.5)	45 (7.5)	0.1529	24 (6.8)	0.1435
Mean (SD)	172	1.8 (1.2)	1.9 (1.2)	0.3331	1.9 (1.1)	0.3007
General anesthesia	173	172 (99.4)	394 (62.4)	< 0.0001	–	–
AHD as first method (only 1st occl. evaluated)	173	151 (87.3)	–	–	–	–
Additional intracranial therapy (before and after Aperio passes)	173	48 (27.7)	133 (21.5)	0.0659	–	–
Pure aspiration (w/o stentretriever)	173	31 (17.9)	41/65 (63.1)	< 0.0001	–	–
Other stentretriever	173	12 (6.9)	29/65 (44.6)	< 0.0001	–	–
Intracranial stenting (with or without PTA)	173	10 (5.8)	15/65 (23.1)	0.0001	–	–
Additional extracranial therapy (PTA and/or stenting)	173	27 (15.7)	–	–	–	–
Aspiration lumen						
Aspiration catheter	173	134 (77.5)	142 (22.6)	< 0.0001	–	–
Balloon guide catheter	173	28 (16.2)	298 (47.3)	< 0.0001	–	–
Aperio sizing						
Undersized	173	4 (2.3)	–	–	–	–
In range	173	31 (17.9)	–	–	–	–
Oversized	173	138 (79.8)	–	–	–	–
Time delays						
Onset to puncture [min], mean (SD)	109	235.7 (168.8)	363.1 (264.5)	< 0.0001	–	–
Puncture to recanalization [min], mean (SD)	173	55.2 (38.6)	78.8 (49.6)	< 0.0001	77 (96.3)	0.0043
*Angiographic, Clinical and Primary Safety Outcome*						
Angiographic Outcome						
mTICI ≥ 2b	173	168 (97.1)	505 (80.3)	< 0.0001	256 (72.5)	< 0.0001
mTICI 0	173	2 (1.2)	–	–	–	–
mTICI 1	173	1 (0.6)	–	–	–	–
mTICI 2a	173	2 (1.2)	79 (12.6)	< 0.0001	–	–
mTICI 2b	173	57 (32.9)	225 (35.8)	0.4910	–	–
mTICI 3	173	111 (64.2)	280 (44.5)	< 0.0001	142 (40.2)	< 0.0001
Embolization to new territory	173	3 (1.7)	20 (4.5)	0.3130	–	–
First pass mTICI ≥ 2b Aperio only*	173	81 (46.8)	–	–	–	–
First pass mTICI 3 Aperio only*	173	60 (34.7)	–	–	–	–
mTICI ≥ 2b Aperio only*	173	146 (84.4)	–	–	–	–
Clinical Outcome						
NIHSS at discharge, mean (SD)	157	3.21 (5.25)	18.1 (18.7)	< 0.0001	–	–
mRS ≤ 2 at 90 days	173	119 (68.8)	277 (47.9)	< 0.0001	132/315 (41.9)	< 0.0001
Primary Safety Outcome						
Periprocedural symptomatic ICH†	173	0 (0)	44 (7.1)	0.0003	35/352 (9.9)	< 0.0001
*Secondary Safety Outcome until 90 days*						
Serious adverse events	173	39 (22.5)	–	–	–	–
Intracranial haemorrhage (symptomatic)	173	5 (2.9)	–	–	–	–
Intracranial haemorrhage (asymptomatic)	173	12 (6.9)	–	–	–	–
Disabling ischemic stroke (MRS 3–6) in the region of the target vessel	173	3 (1.7)	–	–	–	–

*Aperio only means cases without other stentretriever or additional intracranial therapy after the last Aperio pass, †defined as ICH in CT with worsening of NIHSS by ≥ 4 points within 24 h

The table includes statistical comparison with two earlier, otherwise similar registries (NASA and TRACK) for which table reproduction rights were available. See supplementary Table S1 for a more detailed version and the discussion for a more detailed comparison with other previous studies

Several procedural factors (e.g. number of passes, sizing of the AHD, aspiration technique, additional stenting/angioplasty; see Table 1) were assessed. In this context, oversizing of the AHD refers to the situation where the diameter range indicated by the manufacturer for the selected device exceeds the actual vessel diameter in the target region.

Cerebrovascular events that were collected until hospital discharge comprised: Intracranial hemorrhage (symptomatic/asymptomatic), death, transient ischemic attack (TIA) in the region of the target vessel, non-disabling ischemic stroke (mRS 0–2) in the region of the target vessel, disabling ischemic stroke (mRS 3–6) in the region of the target vessel, TIA outside the region of the target vessel, non-disabling ischemic stroke (mRS 0–2) outside the region of the target vessel and disabling ischemic stroke (mRS 3–6) outside the region of the target vessel.

The following additional cerebrovascular events that occurred between discharge and the follow-up interview at 90 days were also recorded: intracranial hemorrhage (symptomatic/asymptomatic), death, TIA, non-disabling ischemic stroke (mRS 0–2), disabling ischemic stroke (mRS 3–6).

Over the whole period of study participation, the following secondary outcome events were recorded for each patient: all-cause mortality at 90 days, serious adverse events, product-related non-serious and serious adverse events, dissection of the target vessel, occlusion of the target vessel, myocardial infarction, severe extracranial hemorrhage (requiring surgical treatment or transfusion).

### Statistical Methods

Although this is a descriptive study, the size of the sample was estimated in such a way that a later metanalytical comparison with other registries or studies on the use of stentretriever systems would be possible with regard to the clinical outcome after 90 days. Based on previous evidence, functional independence (mRS < 3) can roughly be expected in a proportion of 50% of patients after 90 days. Aiming for an error bound of 7.5% (i.e. 15% width of the 95% confidence interval) and expecting a 10% drop-out rate, a sample size of 190 patients was calculated.

The data were processed by an external statistician, commissioned by the sponsor as well as the investigators. Baseline characteristics, technical outcome, clinical outcome and safety related results were analyzed and contrasted with the published results of the TRACK and NASA registries. Student’s t test and F test were used for continuous variables. The χ^2^ and Fisher exact tests were used for categorical variables. Odds ratios and their 95% confidence intervals were calculated with the formulas given by the MedCalc Odds ratio calculator (MedCalc Software Ltd. Odds ratio calculator. https://www.medcalc.org/calc/odds_ratio.php (Version 23.0.6; accessed October 26, 2024)). Further statistical analyses were performed using SPSS (IBM SPSS Statistics, Version 29.0.0.0, RRID:SCR_002865) and MATLAB (MathWorks MATLAB Version: 9.12.0.2009381 (R2022a) Update 4, RRID:SCR_001622). Statistical significance was set at *p* < 0.05 (not corrected for multiple comparisons).

## Results

### Characterization of the Clinical Sample

A total of 190 patients were included in the HYBRID registry (Fig. 1b). Three patients were excluded for reasons that prevented sufficient data collection for the first phase of the study (baseline data, treatment data, hospital follow-up). In this group, one patient withdrew consent and two patients were transferred to another hospital due to local capacity constraints immediately after the procedure. This resulted in a *full analysis set* of 187 patients, for whom all data were available until discharge or transfer to rehabilitation. Among these 187 patients, three patients had protocol deviations (pre-stroke mRS ≥ 3) and 11 patients were lost to follow-up (i.e. 90-day interview data is missing) and were therefore excluded from the *per-protocol clinical outcome analysis*. Reasons for being lost to follow-up was timing of the interview outside the protocol-defined 90-day window (80–100 days) in three patients and eight patients could not be reached for follow-up, neither directly nor via caregivers.

The *per-protocol set* thus contained a total of 173 patients, 156 of whom reached the 90 days follow-up (median: 92, range 80 to 107 days). Seventeen patients died before reaching the 90 days follow-up interview. The whole *per-protocol set* (*n* = 173; including patients who died before the 90 days follow-up) was used for further analysis.

Vessel occlusions were predominantly located in the anterior circulation, with 87.3%, 13.3%, and 0.6% occurring in the MCA, ICA, and ACA, respectively (see Table 1). In the posterior circulation, 4.0 and 1.7% of occlusions were found in the BA and PCA, respectively.

Average vessel diameters were 2.5 ± 0.8 mm at the proximal thrombus boundary and 1.8 ± 0.5 mm at the distal thrombus boundary. Aspiration was performed using an aspiration catheter in 77.5% of procedures, a balloon guide catheter in 16.2%, a non-balloon guide catheter in 63.6%, and a long sheath in 5.2%, respectively. As multiple aspiration techniques were often used in combination, the percentages exceed 100%. Importantly, at least one form of aspiration was applied in all procedures. Regarding the procedural sequence, the Aperio Hybrid Device (AHD) was used in 151 patients (87.3%) as the first-line device, in 20 patients (11.6%) as a rescue device following unsuccessful primary aspiration without prior stent retriever, and in 2 patients (1.2%) after failure of a different stent retriever. Additional stenting (with or without PTA) was performed in 5.8% of cases intracranially and in 15.7% extracranially. Average number of passes was 1.8 ± 1.2.

### Technical Success

Technical success (mTICI ≥ 2b) was achieved with the AHD alone in 84.4% of patients. AHD alone means cases without other stentretriever or additional intracranial therapy such as pure aspiration, after the last AHD pass. Moreover, a first-pass mTICI 3 reperfusion with the AHD alone was achieved in 34.7% of cases.

The overall mTICI ≥ 2b recanalization rate (when including combined intracranial recanalization techniques, e.g. with pure aspiration, alternative stent retrievers, or intracranial stenting) was 97.1% (see Table 1). A first pass success (mTICI ≥ 2b after a single pass) with the AHD was achieved in 47.4% of the patients.

In 23 patients (13.3%), additional intracranial techniques were employed after the final use of the Aperio Hybrid Device (AHD) to achieve successful recanalization. These adjunctive measures were applied either due to insufficient initial reperfusion or to further improve the reperfusion grade (e.g., from mTICI 2b to 3). Among these cases, pure aspiration was used in 10 (5.8%) patients, intracranial percutaneous transluminal angioplasty with stenting in 10 (5.8%) patients, and an alternative stent retriever in 10 (5.8%) patients; in several patients, a combination of these techniques was applied.

**Fig. 2:**
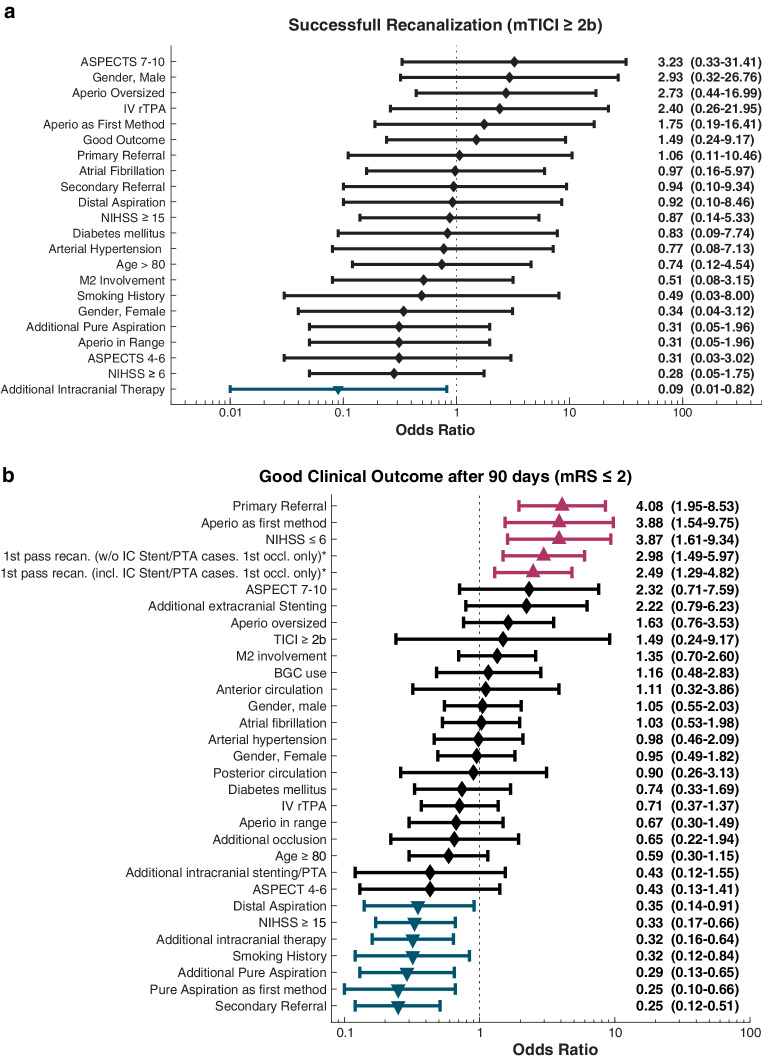
**a** Summary of potential mediators of technical recanalization success. Odds ratios (confidence intervals in parentheses, blue: significant negative association, not corrected for multiple comparisons). Figure design was inspired by the forest plot in the publication of the results of the NASA trial [14]. **b** Summary of potential mediators of good clinical outcomes. Odds ratios (confidence intervals in parentheses; red: significant positive association, blue: significant negative association, not corrected for multiple comparisons). See Table 2 for absolute patient numbers for individual data points. Figure design was inspired by the forest plot in the publication of the results of the NASA trial [14]

Several factors were analyzed for their influence on technical success by calculating the odds ratio and the 95% confidence interval, respectively (Fig. 2a). Additional intracranial therapy (pure aspiration, other stentretriever, intracranial stenting and/or angioplasty) was associated with a reduced odds ratio (0.04, 95% CI 0.00–0.35) for technical success.

Supplementary data on angiographic outcome in the *full analysis set* (including those patients who were lost to follow-up) is presented in supplementary Table S2.

### Clinical Outcome

**Fig. 3:**
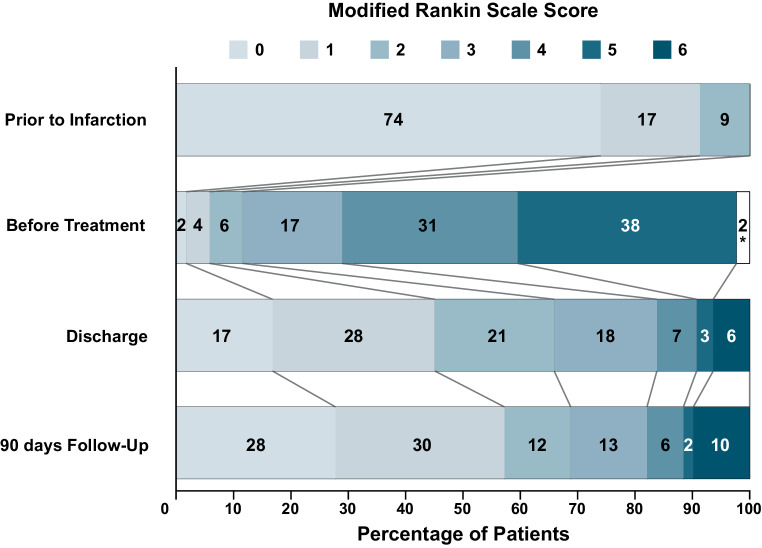
Disability or dependence at different timepoints related to stroke treatment. Percentages do not add to 100% due to rounding, *missing data

Mean NIHSS at time of discharge was 3.2 ± SD 5.3. Good clinical outcome at 90 days (mRS ≤ 2) was observed in 68.8% of patients (see Fig. 3 and Table 2 for an overview of the mRS distribution).

**Table 2:** Distribution of clinical outome at different time points.

Clinical Outcome	mRS 0	mRS 1	mRS 2	mRS 3	mRS 4	mRS 5	mRS 6	Missing data
	*n* (%)	*n* (%)	*n* (%)	*n* (%)	*n* (%)	*n* (%)	*n* (%)	*n* (%)
Prior to Infarction	128 (74.0)	30 (17.3)	15 (8.7)	0 (0)	0 (0)	0 (0)	0 (0)	0 (0)
Before Treatment	3 (1.7)	7 (4.0)	10 (5.8)	30 (17.3)	53 (30.6)	66 (38.2)	0 (0)	4 (2.3)
Discharge	29 (16.8)	49 (28.3)	36 (20.8)	31 (17.9)	12 (6.9)	5 (2.9)	11 (6.4)	0 (0)
90 days Follow-Up	48 (27.7)	51 (29.5)	20 (11.6)	23 (13.3)	11 (6.4)	3 (1.7)	17 (9.8)	0 (0)

The table shows absolute patient numbers and percentage values (in relation to the per-protocol-set; n = 173) for the distribution of the clinical outcome over different ratings on the modified Rankin Scale (mRS) at four different time points of the study

‘Before Treatment’ mRS values were missing for four patients

Several factors were analyzed for their association with good clinical outcome by calculating the odds ratio and the 95% confidence interval, respectively (Fig. 2b). Increased odds ratios were found for primary referral (4.08, 95% CI 1.95–8.53), use of the AHD as the first method (3.88, 95% CI 1.54–9.75), NIHSS ≤ 6 (3.87, 95% CI 1.61–9.34), first pass recanalizations without intracranial stent/angioplasty cases (2.98, 95% CI 1.49–5.97) and first pass recanalizations without intracranial stent/angioplasty cases (2.49, 95% CI 1.29–4.82).

Significantly decreased odds ratios were found for distal aspiration (0.35, 95% CI 0.14–0.91), NIHSS ≥ 15 (0.33, 95% CI 0.17–0.66), additional intracranial therapy (0.32, 95% CI 0.16–0.64), smoking history (0.32, 95% CI 0.12–0.84), additional pure aspiration (0.29, 95% CI 0.13–0.65), pure aspiration as the first method (0.25, 95% CI 0.10–0.66) and secondary referral (0.25, 95% CI 0.12–0.51).

Supplementary data on clinical outcome in the *full analysis set* (including those patients who were lost to follow-up) is presented in supplementary Table S3.

### Safety Outcome

No periprocedural sICH (i.e. < 24 h after the procedure) occurred in HYBRID.

However, 17 intracranial hemorrhages which did not meet the criteria of a periprocedural sICH were observed until hospital discharge. Subarachnoid hemorrhage (SAH) occurred in three cases (1.7%). Two of these were symptomatic and one was asymptomatic. Among the symptomatic cases, one involved an oversized stent retriever and the other was sized within the recommended range; the asymptomatic SAH occurred with a device that was undersized relative to the target vessel.

No further ICH were reported at 90 days. Five of these were classified as late sICH, while the rest of the bleedings remained clinical silent. In total, 39 serious adverse events were reported until 90 days, of which none was considered device-related. Further secondary safety outcomes are reported in Table 1.

## Discussion

In this prospective, multicentric, externally monitored register including adult patients with AIS predominantly in the anterior circulation, relatively low pre-stroke disability and relatively low early signs of infarction, treated with the AHD, we observed relatively high technical success rates with the AHD only and very high technical success rates in combination with other devices. However, in less than half of the cases mTICI ≥ 2b was achieved as a first pass effect with the AHD. Both, initial safety outcomes and clinical outcomes at 90 days were favorable.

### Technical Success

The recanalization rate in our registry study was relatively high, with an mTICI ≥ 2b outcome achieved in 84.4% of cases with the AHD alone—without the need for additional intracranial therapies (after the last pass of the AHD) such as pure aspiration, alternative stent retrievers, or intracranial stenting. This compares favorably to the successful recanalization (mTICI ≥ 2b) rates reported in the HERMES collaboration meta-analysis (71%) [30], the data from the STRATIS registry (87.9%) [31] , the TRACK multicenter Registry (80.3%) [13] , NASA [14] (72.5%), ARISE II Study (80.2%) [32] and EXCELLENT Registry (94.5%) [33]. Accordingly, complete recanalizations (mTICI = 3) were observed in 64.2% of HYBRID patients, compared to 44.5% (TRACK) [13] and 40.2% (NASA) [14]. This discrepancy may be partially attributed to advancements in device/setup technology since data collection of the five major trials included in the HERMES meta-analysis (prior to 2015) as supported by a longitudinal analysis of MR CLEAN registry data [34]. Additionally, 99.4% of patients in our registry study were treated under general anesthesia, a factor that may have contributed to the high recanalization rate [35].

When including 23 cases with other techniques used for successful intracranial recanalization after the last pass of the AHD (e.g. pure aspiration, alternative stent retrievers, or intracranial stenting), the overall mTICI ≥ 2b recanalization rate was 97.1%, reflecting the practical application of AHD in combination with other techniques—showing that its use does not preclude successful rescue interventions when needed.

Additionally, our study identified a positive association between successful recanalization (mTICI ≥ 2b) and AHD oversizing (OR 2.73; see Fig. 2a). In this context, oversizing refers to the situation where the diameter range indicated by the manufacturer for the selected AHD exceeds the actual vessel diameter in the target region. This size-related finding is consistent with a sub-analysis of the STRATIS Registry, which examined the impact of stentretriever size on angiographic outcomes. Specifically, longer stentretrievers (4 × 40 mm) achieved the highest first-pass effect (FPE) and modified FPE compared to larger-diameter or shorter retrievers [36]. Similarly, Candel et al. reported that stentretrievers with greater length or nominal diameter yielded higher rates of complete and successful FPE, as well as improved recanalization success, particularly in thrombectomy for acute M1 occlusions [37].

Furthermore, successful recanalization was associated with the selection of AHD as the first-line approach (OR 1.75; see Fig. 2a). 

### Clinical Outcomes

Overall, the mean NIHSS at discharge in the HYBRID cohort was relatively low (3.2 ± 5.3) compared to TRACK [13] (18.1 ± 18.7; see Table 1). A good clinical outcome at 90 days (mRS ≤ 2) was achieved in 68.8% of HYBRID patients, exceeding the outcomes reported in HERMES (46%) [30], MR CLEAN registry (37.9%) [38], STRATIS (56.5%) [31] , NASA(42%) [14], the TREVO registry/TRACK (47.9%) [13] , EXCELLENT (46.8%) [33] , and ARISE II (67%) [32]. In the subgroup of patients with medium and distal vessel occlusions (MeVO/DVO) (*n* = 45), 77.7% achieved a good clinical outcome at 90 days (mRS ≤ 2), surpassing both the overall HYBRID cohort and the outcomes reported in the DISTAL (56.5%) [39] and ESCAPE MeVO trials (54%) [40].

### Safety Outcomes

In our study, no periprocedural symptomatic intracranial hemorrhage (sICH) was reported. In contrast, sICH was observed in 7.1% of patients in the TRACK study [13], 9.9% in the NASA registry [14], 1.4% in the STRATIS registry [36], 1.6% in the EXCELLENT registry [33], 4.4% in the HERMES collaboration meta-analysis [30], 2% in the SWIFT cohort [41], 5.6% in the ARISE II study [32], and 7% in the TREVO 2 trial [42]. Notably, no periprocedural sICH occurred in cases of AHD oversizing, which accounted for 79.8% of cases. This indicates that the application of the AHD was safely feasible. The incidence of sICH in our HYBRID cohort was therefore significantly lower than in the studies and registries mentioned above. This finding is particularly noteworthy given that medium and distal vessel occlusions were also included. However, this discrepancy may be partly attributable to a potential underestimation of sICH in our study, as early mortality cases without subsequent imaging precluded a definitive distinction between stroke-related and procedure-related events, such as undetected sICH, as the cause of death.

### Comparibility with Other Stentretrievers and Studies

Comparisons with other stentretrievers are limited to post-hoc or exploratory analyses. These exploratory statistical analyses include comparisons with two previous registries (TRACK [13] and NASA [14]) as surrogates for the state-of-the art for conventional stentretriever designs. Direct comparability is, however, limited by less strict inclusion criteria of those previous studies, mainly regarding pre-stroke disability [13, 14] as well as clinical baseline characteristics and procedural details (see Table 1): In particular, HYBRID patients were older, while cardiovascular risk factors were less frequent. The proportion of patients who received intravenous tissue plasminogen activator (tPA) was lower in HYBRID than in TRACK (51.2%). Initial mean NIHSS was lower in HYBRID (11.3 ± 7.4) than in both TRACK (17.4 ± 6.7) and NASA (18.1 ± 6.6). ICA occlusions were less frequent than in NASA, while MCA occlusions were more frequent in HYBRID than in both TRACK and NASA. Only 6.9% of patients in HYBRID had posterior circulation occlusions, which was less frequent than in TRACK (12.7%). General anesthesia was used in nearly all patients in HYBRID,while this was the case in only 62.4% of patients in TRACK. The use of balloon guide catheters (BCG) was less common in HYBRID (16.2%) than in TRACK (47.3%), while aspiration catheters were used more frequently in HYBRID. Time delays were shorter in HYBRID compared to TRACK and NASA. Furthermore, NASA adopted the original TICI rather than the mTICI score [14].

### Limitations

Technical success rating was based on the older mTICI definition [26] without a 2c category (i.e. near-complete recanalization). More recently, it has been questioned, whether this missing TICI 2c/3 should define technical success as a lower bound rather than mTICI 2b [43–45].

Another limitation of the study is that imaging and clinical endpoints were not assessed centrally. Instead, evaluations were performed by the investigators at the respective study sites. The absence of an independent core lab or centralized adjudication may introduce interobserver variability and limit the objectivity of the reported outcomes [46, 47].

Given the single-arm design and the lack of prespecified testable hypotheses limits this report mainly to a descriptive statistical approach, supplemented by post-hoc or exploratory analyses.

Although screening logs were maintained to ensure the most consecutive and unbiased patient inclusion possible, potential selection bias cannot be fully excluded.

Analysis and interpretation of the potential impact of stentretriever sizing [36, 48], especially the relatively high rate of diameter oversizing in relation to the target vessel diameter are limited by a potential site bias, caused by the treating neurointerventionalists’ sizing preferences. Otherwise, potential site biases were addressed by the HYBRID study’s multicenter design and external monitoring.

The high technical success rate and relatively good clinical outcomes observed in this study compared with previous registries [13, 14] do not necessarily reflect device-specific benefits of the AHD or the other devices and techniques used to treat patients included in this PMCF registry. In addition to possible reasons discussed in the respective sections, they might in part be related to a selection bias owing to the inclusion being restricted to patients with relatively low pre-stroke morbidity, as defined by the inclusion criteria. Results can thus not be directly translated to all real-world treatment scenarios for people with AIS resulting from large vessel occlusion [49].

### Conclusions

The AHD current-generation stentretriever was both effective and safe. Primary safety (no periprocedural sICH even despite a high stentretriever oversizing rate) and clinical outcomes surpassed earlier registries on different stentretrievers of the same and earlier generations. However, it cannot be distinguished, whether this reflects true device characteristics, refined general endovascular stroke therapy setup and techniques or selection biases which are inherent to PMCF studies in general.

## Supplementary Information

Tab. S1 Detailed information on baseline clinical and imaging characterization, type of occlusion and treatment. The table shows absolute patient numbers and percentage values for the distribution of the clinical outcome over different ratings on the modified Rankin Scale (mRS) at four different time points of the study for all 187 patients, for whom all data was available until discharge (full analysis set).

Tab. S2 Angiographic outcome for the full analysis set. The table shows absolute patient numbers and percentage values for the distribution of the clinical outcome over different ratings on the modified Rankin Scale (mRS) at four different time points of the study for all 187 patients, for whom all data was available until discharge (full analysis set).

Tab. S3 Distribution of clinical outome at different time points for the full analysis set. ‘Before Treatment’ mRS values were missing for four patients.

## Data Availability

Due to German data protection regulations and to safeguard subject confidentiality, data on the level of individual participants cannot be made available (no participant consent for data sharing). Data ownership: Acandis GmbH, Pforzheim, Germany.
