# Supplementary material for: Safety, Technical and Clinical Success of the Aperio Hybrid Thrombectomy Device in Acute Ischemic Stroke, a Prospective Post-market Clinical Follow-up Study (HYBRID)
Source: Clin Neuroradiol. 2025 Oct 23;36(1):203–16. doi: 10.1007/s00062-025-01578-5 (PMC13009069; doi:10.1007/s00062-025-01578-5)
Supplement: Supplementary file 1 — Tab. S1 Detailed information on baseline clinical and imaging characterization, type of occlusion and treatment. The table shows absolute patient numbers and percentage values for the distribution of the clinical outcome over different ratings on the modified Rankin Scale (mRS) at four different time points of the study for all 187 patients, for whom all data was available until discharge (full analysis set). [file 62_2025_1578_MOESM1_ESM.pdf]

	Aperio Hybrid (n=173)		TRACK (n=629)		NASA (n=354)	
	Data Available	N(%)	N(%)	p Value Hybrid versus TRACK	N(%)	p Value Hybrid versus NASA
Age [years], mean (SD)	172	73.2 (12.1)	66.1 (14.8)	< 0.0001	67.3 (15.2)	< 0.0001
Sex (female)	173	101 (58.4)	305 (48.3)	0.0212	176 (49.7)	0.0614
Arterial hypertension	173	131 (75.7)	473 (75.0)	0.8875	271 (76.6)	0.8332
Prior stroke	173	27 (15.6)	-	-	-	-
Artrial fibrillation	173	68 (39.3)	247 (39.2)	0.9928	148 (41.8)	0.5835
Diabetes mellitus	173	30 (17.3)	161 (25.5)	0.0240	87 (24.6)	0.0606
Hyperlipidemia	173	58 (33.5)	314 (49.8)	0.0001	182 (51.4)	0.0001
Coronary heart disease	173	28 (16.2)	146 (29.4)	0.0471	111 (31.4)	0.0002
Smoking history	173	38 (22.0)	154 (24.5)	0.4919	108 (30.5)	0.0396
Current smoker	173	27 (15.6)	-	-	-	-
Former smoker	173	11 (6.4)	-	-	-	-
Antiplatelets	173	45 (26.0)	-	-	-	-
ASA	173	42 (24.3)	-	-	-	-
Clopidogrel	173	5 (2.9)	-	-	-	-
other Antiplatelet	173	1 (0.6)	-	-	-	-
Anticoagulation	173	41 (23.7)	-	-	-	-
Statins	173	57 (32.9)	-	-	-	-
Antihypertensives	173	122 (70.5)	-	-	-	-
Antidiabetics	173	14 (8.1)	-	-	-	-
IV tPA	173	64 (37.0)	321 (51.2)	0.0011	-	-
mRS prior to Infarction			-	-	-	-
0	173	128 (74.0)	-	-	-	-
1	173	30 (17.3)	-	-	-	-
2	173	15 (8.7)	-	-	-	-
mRS before treatment (missing data in 4 patients)			-	-	-	-
0	169	3 (1.8)	-	-	-	-
1	169	7 (4.1)	-	-	-	-
2	169	10 (5.9)	-	-	-	-
3	169	30 (17.8)	-	-	-	-
4	169	53 (31.4)	-	-	-	-
5	169	66 (39.1)	-	-	-	-
Initial NIHSS, median (IQR)	169	10 (5-17)	-	-	-	-
Initial NIHSS, mean (SD)	169	11.3 (7.4)	17.4 (6.7)	< 0.0001	18.1 (6.6)	< 0.0001
Initial systolic BP [mmHg], mean (SD)	149	163.6 (29.0)	144.9 (26.6)	< 0.0001	-	-
Initial diastolic BP [mmHg], mean (SD)	148	88.4 (18.4)	78.2 (19.2)	< 0.0001	-	-

In-house	173	2 (1.2)	-	-	-	-
Primary referral	173	130 (75.1)	-	-	-	-
Secondary referral	173	41 (23.7)	-	-	-	-
Left	173	73 (42.2)	-	-	-	-
Right	173	92 (53.2)	-	-	-	-
Midline	173	8 (4.6)	-	-	-	-
ACA	173	1 (0.6)	-	-	-	-
MCA	173	137 (79.2)	-	-	-	-
MCA M2 or more distal	173	46 (26.6%)	-	-	-	-
ICA/MCA	173	10 (5.8)	-	-	-	-
ICA/MCA/ACA	173	4 (2.3)	-	-	-	-
ICA	173	9 (5.2)	-	-	-	-
MCA combined	173	151 (87.3)	434 (68.9)	< 0.0001	197 (55.6)	< 0.0001
ICA combined	173	23 (13.3)	100 (15.9)	0.4000	82 (23.2)	0.0077
VA (V4)	173	1 (0.6)	-	-	-	-
BA	173	7 (4.0)	-	-	36 (10.2)	0.0159
BA/PCA	173	1 (0.6)	-	-	-	-
PCA	173	3 (1.7)	-	-	-	-
Anterior circulation	173	161 (93.1)	546 (86.7)	0.0241	-	-
Posterior circulation	173	12 (6.9)	84 (12.7)	0.0213	-	-
Vessel diam. (prox. thrombus boundary)	173	2.5 (0.8)	-	-	-	-
Vessel diam. (dist. thrombus boundary)	173	1.8 (0.5)	-	-	-	-
Length of thrombus [mm], mean (SD)	173	9.7 (11.2)	-	-	-	-
More than one occlusion treated	173	15 (8.7)	-	-	-	-
**ASPECTS**						
4	158	1 (0.6)	-	-	-	-
5	158	4 (2.5)	-	-	-	-
6	158	7 (4.4)	-	-	-	-
7	158	21 (13.3)	-	-	-	-
8	158	28 (17.7)	-	-	-	-
9	158	37 (23.4)	-	-	-	-
10	158	60 (38.0)	-	-	-	-
**Number of passes (only 1st occl. evaluated, other devices included)**						
1	172	95 (55.2)	272 (54.6)	0.0052	172 (48.6)	0.1527
2	172	39 (22.7)	167 (28.2)	0.3028	94 (26.6)	0.3369
3	172	20 (11.6)	112 (18.8)	0.0529	64 (18.1)	0.0581
>3	172	18 (10.5)	45 (7.5)	0.1529	24 (6.8)	0.1435
median (IQR)	172	1 (1-2)	-	-	2 (1-2)	-

mean (SD)	172	1.8 (1.2)	1.9 (1.2)	0.3331	1.9 (1.1)	0.3007
**General anesthesia**	173	172 (99.4)	394 (62.4)	< 0.0001	-	-
**First method (only 1st occl. evaluated)**						
Aperio	173	151 (87.3)	-	-	-	-
Pure aspiration	173	20 (11.6)	-	-	-	-
Other Stentretriever	173	2 (1.2)	-	-	-	-
**Additional intracranial therapy (before and after Aperio passes)**	173	48 (27.7)	133 (21.5)	0.0659	-	-
Pure aspiration (w/o stentretriever)	173	31 (17.9)	41/65 (63.1)	< 0.0001	-	-
Other stentretriever	173	12 (6.9)	29/65 (44.6)	< 0.0001	-	-
Intracranial stenting (with or without PTA)	173	10 (5.8)	15/65 (23.1)	0.0001	-	-
**Additional intracranial therapy (after last Aperio pass)**	173	26 (16.2)	-	-	-	-
Pure aspiration (w/o stentretriever)	173	10 (5.8)	-	-	-	-
Other stentretriever	173	10 (5.8)	-	-	-	-
Intracranial stenting (with or without PTA)	173	10 (5.8)	-	-	-	-
**Additional extracranial therapy (PTA and/or stenting)**	173	27 (15.7)	-	-	-	-
**Aspiration lumen (only 1st occl. evaluated)**						
Aspiration catheter	173	134 (77.5)	142 (22.6)	< 0.0001	-	-
Balloon guide catheter	173	28 (16.2)	298 (47.3)	< 0.0001	-	-
Non-balloon guide catheter	173	110 (63.6)	-	-	-	-
Long sheath	173	9 (5.2)	-	-	-	-
**Aspiration method**						
Pump	173	62 (35.8)	-	-	-	-
Syringe	173	111 (64.2)	-	-	-	-
**Aperio sizing**						
undersized	173	4 (2.3)	-	-	-	-
in range	173	31 (17.9)	-	-	-	-
oversized	173	138 (79.8)	-	-	-	-
**Time delays**						
Onset to puncture [min], mean (SD)	109	235.7 (168.8)	363.1 (264.5)	< 0.0001	-	-
Puncture to recanalization [min], mean (SD)	173	55.2 (38.6)	78.8 (49.6)	< 0.0001	77 (96.3)	0.0043
**Angiographic Outcome**						
mTICI ≥ 2b	173	168 (97.1)	505 (80.3)	< 0.0001	256 (72.5)	< 0.0001
mTICI 0	173	2 (1.2)	-	-	-	-
mTICI 1	173	1 (0.6)	-	-	-	-
mTICI 2a	173	2 (1.2)	79 (12.6)	< 0.0001	-	-
mTICI 2b	173	57 (32.9)	225 (35.8)	0.4910	-	-
mTICI 3	173	111 (64.2)	280 (44.5)	< 0.0001	142 (40.2)	< 0.0001
Embolization to new territory	173	3 (1.7)	20 (4.5)	0.3130	-	-
First pass success with Aperio*	173	81 (46.8)	-	-	-	-
mTICI ≥ 2b with Aperio only*	173	146 (84.4)	-	-	-	-
**Clinical Outcome**						

NIHSS at discharge, median (IQR)	157	2 (1-3)	-	-	-	-
NIHSS at discharge, mean (SD)	157	3.21 (5.25)	18.1 (18.7)	< 0.0001	-	-
mRS ≤2 at 90 days	173	119 (68.8)	277 (47.9)	< 0.0001	132/315 (41.9)	< 0.0001
mRS at 90 days 0	173	48 (27.7)	-	-	-	-
mRS at 90 days 1	173	51 (29.5)	-	-	-	-
mRS at 90 days 2	173	20 (11.6)	-	-	-	-
mRS at 90 days 3	173	23 (13.3)	-	-	-	-
mRS at 90 days 4	173	11 (6.4)	-	-	-	-
mRS at 90 days 5	173	3 (1.7)	-	-	-	-
mRS at 90 days 6 (Mortality)	173	17 (9.8)	-	-	-	-
**Primary Safety Outcome**						
Periprocedural symptomatic ICH†	173	0 (0)	44 (7.1)	0.0003	35/352 (9.9)	< 0.0001
Serious adverse events	173	39 (22.5)	-	-	-	-
Device related non-serious adverse events	173	0 (0)	-	-	-	-
Dissection of the target vessel	173	0 (0)	-	-	-	-
Occlusion of the target vessel	173	0 (0)	-	-	-	-
Myocardial infarction	173	1 (0.6)	-	-	-	-
Severe extracranial haemorrhage (req. surgery or transfusion)	173	1 (0.6)	-	-	-	-
Intracranial haemorrhage (symptomatic / asymptomatic)	173	17 (9.8)	-	-	-	-
Intracranial haemorrhage (symptomatic)	173	5 (2.9)	-	-	-	-
Intracranial haemorrhage (asymptomatic)	173	12 (6.9)	-	-	-	-
TIA in the region of the target vessel	173	0 (0)	-	-	-	-
Non-disabling ischemic stroke (MRS 0-2) in the region of the target vessel	173	0 (0)	-	-	-	-
Disabling ischemic stroke (MRS 3-6) in the region of the target vessel	173	3 (1.7)	-	-	-	-
TIA outside the region of the target vessel	173	1 (0.6)	-	-	-	-
Non-disabling ischemic stroke (MRS 0-2) outside the region of the target vessel	173	0 (0)	-	-	-	-
Disabling ischemic stroke (MRS 3-6) outside the region of the target vessel	173	2 (1.2)	-	-	-	-

*Aperio only means cases without other stentretriever or additional intracranial therapy after the last Aperio pass

†defined as ICH in CT with worsening of NIHSS by ≥ 4 points within 24 h
