# Supplementary material for: Safety, Technical and Clinical Success of the Aperio Hybrid Thrombectomy Device in Acute Ischemic Stroke, a Prospective Post-market Clinical Follow-up Study (HYBRID)
Source: Clin Neuroradiol. 2025 Oct 23;36(1):203–16. doi: 10.1007/s00062-025-01578-5 (PMC13009069; doi:10.1007/s00062-025-01578-5)
Supplement: Supplementary file 2 — Tab. S2 Angiographic outcome for the full analysis set. The table shows absolute patient numbers and percentage values for the distribution of the clinical outcome over different ratings on the modified Rankin Scale (mRS) at four different time points of the study for all 187 patients, for whom all data was available until discharge (full analysis set). [file 62_2025_1578_MOESM2_ESM.docx]

| Angiographic Outcome | mTICI 0 | mTICI 1 | mTICI 2a | mTICI 2b | mTICI 3 |
| --- | --- | --- | --- | --- | --- |
|  | n (%) | n (%) | n (%) | n (%) | n (%) |
| Full Analysis Set (n=187) | 2 (1.1) | 1 (0.5) | 3 (1.6) | 63 (33.7) | 118 (63.1) |

**Tab. S2 – Angiographic outcome for the full analysis set.** The table shows absolute patient numbers and percentage values for the distribution of the clinical outcome over different ratings on the modified Rankin Scale (mRS) at four different time points of the study for all 187 patients, for whom all data was available until discharge (full analysis set).
