# Supplementary material for: Safety, Technical and Clinical Success of the Aperio Hybrid Thrombectomy Device in Acute Ischemic Stroke, a Prospective Post-market Clinical Follow-up Study (HYBRID)
Source: Clin Neuroradiol. 2025 Oct 23;36(1):203–16. doi: 10.1007/s00062-025-01578-5 (PMC13009069; doi:10.1007/s00062-025-01578-5)
Supplement: Supplementary file 3 — Tab. S3 Distribution of clinical outome at different time points for the full analysis set. ‘Before Treatment’ mRS values were missing for four patients. [file 62_2025_1578_MOESM3_ESM.docx]

| Clinical Outcome | mRS 0 | mRS 1 | mRS 2 | mRS 3 | mRS 4 | mRS 5 | mRS 6 | missing data |
| --- | --- | --- | --- | --- | --- | --- | --- | --- |
|  | n (%) | n (%) | n (%) | n (%) | n (%) | n (%) | n (%) | n (%) |
| Prior to Infarction | 135 (72.2) | 31 (16.6) | 19(10.2) | 1 (0.5) | 0 (0) | 1 (0.5) | 0 (0) | 0 (0) |
| Before Treatment | 3 (1.6) | 8 (4.3) | 12 (6.4) | 31 (16.6) | 56 (29.9) | 73 (39.0) | 0 (0) | 4 (2.1) |
| Discharge | 30 (16.0) | 52 (27.8) | 38 (20.3) | 33 (17.6) | 14 (7.5) | 8 (4.3) | 12 (6.4) | 0 (0) |

**Tab. S3 – Distribution of clinical outome at different time points for the full analysis set.** The table shows absolute patient numbers and percentage values for the distribution of the clinical outcome over different ratings on the modified Rankin Scale (mRS) at four different time points of the study for all 187 patients, for whom all data was available until discharge (full analysis set). ‘Before Treatment’ mRS values were missing for four patients.
